# Renal ischemia/reperfusion injury; from pathophysiology to treatment

**DOI:** 10.12861/jrip.2015.06

**Published:** 2015-06-01

**Authors:** Maryam Malek, Mehdi Nematbakhsh

**Affiliations:** ^1^Water and Electrolytes Research Center/Department of Physiology, Isfahan University of Medical Sciences, Isfahan, Iran; ^2^Isfahan MN Institute of Basic and Applied Sciences Research, Isfahan , Iran

**Keywords:** Renal injury, Ischemia/reperfusion, Acute kidney injury, Reactive oxygen species

## Abstract

Ischemia/reperfusion injury (IRI) is caused by a sudden temporary impairment of the blood flow to the particular organ. IRI usually is associated with a robust inflammatory and oxidative stress response to hypoxia and reperfusion which disturbs the organ function. Renal IR induced acute kidney injury (AKI) contributes to high morbidity and mortality rate in a wide range of injuries. Although the pathophysiology of IRI is not completely understood, several important mechanisms resulting in kidney failure have been mentioned. In ischemic kidney and subsequent of re-oxygenation, generation of reactive oxygen species (ROS) at reperfusion phase initiates a cascade of deleterious cellular responses leading to inflammation, cell death, and acute kidney failure. Better understanding of the cellular pathophysiological mechanisms underlying kidney injury will hopefully result in the design of more targeted therapies to prevent and treatment the injury. In this review, we summarize some important potential mechanisms and therapeutic approaches in renal IRI.

**Implication for health policy/practice/research/medical education:**

Renal injury associated with ischemia/reperfusion results from a dynamic process involving inflammation and some mediators in a complex interaction. Formation of oxidative stress and lipid peroxidation seems to be major factors which promotes the inflammation process during ischemia/reperfusion injury. A better understanding of the pathophysiology and therapeutic approach underlying the functional defects found in ischemic acute renal failure will also require that we take into account the complexity of illness.

## Introduction

Ischemia/reperfusion injury (IRI) is characterized by restriction of blood supply to an organ followed by restoration of blood flow and re-oxygenation. The inevitable injuries may occur after infarction, sepsis and organ transplantation and this phenomena exacerbate tissue damage by initiating an inflammatory cascade including reactive oxygen species (ROS), cytokines, chemokines, and leukocytes activation (1,2). In the kidney, IRI contributes to pathological conditions called acute kidney injury (AKI) that is a clinical syndrome with rapid kidney dysfunction and high mortality rates (3,4). The pathophysiology of IRI in kidney is very complex but some pathological pathways such as activation of neutrophils, release of reactive oxygen species and other inflammatory mediators including adhesion molecules and a variety of cytokines are involved. Studies have demonstrated the beneficial effects of different agents in combat with IRI, for example, doxycycline through reducing the level of pro-inflammatory cytokines (5,6), leptin by decreasing tumor necrosis factor alpha (TNF-α) level and increasing nitrite level (7), levosimendan through antioxidant and NO-related mechanisms (8), iloprost by suppression of lipid peroxidation and (9) ascorbic acid via free radical scavenging and antioxidant activities (10).

## Materials and Methods

For this review, we used a variety of sources by searching through PubMed, Embase, Scopus and directory of open access journals (DOAJ). The search was performed by using combinations of the following key words and or their equivalents; renal injury, ischemia/reperfusion, AKI, reactive oxygen species. Manuscripts published in English as full-text articles and or as abstracts were included in the study.

## Inflammation, leukocytes and adhesion molecules

Inflammation as a common abnormality in kidney IRI seems to link the various cell types and playing an important role in its pathophysiology. Renal IRI triggers an inflammatory cascade that involved in more renal damages, so inhibition of inflammatory responses is a therapeutic approach to protect renal tissue (11,12). Chemokines are major mediators of the inflammation that regulate pro-inflammatory cytokine, adhesion molecule expression, leukocyte infiltration and activation (13). Pro-inflammatory cytokines and cytokines such as interleukin 6 (IL6) and TNFα play a major role in renal dysfunction of IRI (14-16). Activation of Janus kinase/signal transducer and activator of transcription (JAK/STAT) pathway mediated many pro-inflammatory cytokines that involved in progression of renal IRI (17). Dexmedetomidine (a highly selective α2-adrenoreceptor agonist) has a cytoprotective effect against renal IRI by inhibiting the phosphorylation of JAK/STAT proteins, reducing IL6 and TNFα that indicating its anti-inflammatory effects (18-22).

Inflammatory mediators, ROS and cell adhesion molecules – intracellular adhesion molecule-1 (ICM-1) and P-selectin – recruit leukocytes and neutrophil infiltration into post ischemic tissue, then leads to enhanced leukocyte – endothelial interactions, which can promote injury, swelling of the endothelial cell and physically impede blood flow (23-26). Administration of ICM-1 antibody and the kidneys of ICM-1 knockout were protected from IRI in mice (27,28). Takada et al. showed that soluble P-selectin ligand attenuates post-ischemic neutrophil infiltration and injury by inhibiting the binding between P-selectin and leukotriene aggregation (26). In addition Patel et al. showed that endogenous 5-lipoxygenase metabolites enhanced the degree of renal injury, dysfunction, and inflammation caused by kidney IRI via expression of adhesion molecules while in 5-lipoxygenase knockout mice the renal IRI were ameliorated (29). Involvement of the inflammatory leukotriene pathway in IRI has been demonstrated in acute and chronic renal failure (29-32). Activation of leukocytes, especially neutrophils have an important role in the development of renal IRI (33,34). Neutrophils releases ROS, cytokines, proteases and other mediators that exceed IRI (35). Montelukast, zafirlukast and cysteinyl leukotriene receptor blockers, demonstrated protective effects on renal IRI through inhibition of neutrophil infiltration, suppression of adhesion molecules and lipid peroxidation (30,36).

Many agents also have protecting effects on IRI through anti-inflammatory properties that mention in below. Nicotin is an anti-inflammatory cholinergic agonist, protect renal function after IRI by suppressing neutrophil infiltration, chemokines release and inflammation through an α7 nicotine acetylcholine receptor (α7nAchR) (37,38). These renoprotective effects also reported in vagotomized animals and suggest that cholinergic agonists act directly within the kidney (38).

Celastrol is a bioactive ingredient of chines herb “*Tripterygium wilfordii*” with anti-inflammatory and antioxidant activities that used in treating auto-immune diseases and chronic nephritis. Celastrol protect IRI by inhibiting neutrophil infiltration, lipid peroxidation and suppressing the induction of pro-inflammatory mediators synthesizes such as cyclooxygenase-2 (COX2) presumably by suppressing nuclear factor κB (NF-κB) signaling pathway (39). Besides controversial studies reported that celastrol promoted the kidney injury in renal IRI by upregulation of COX2 and prostaglandin E2 (PGE2) synthesis (40). As a conclusion it seems that inflammation, leukocytes and adhesion molecules are seriously involved in IRI process, and any agents that suppress inflammation process, or inhibit leukocytes and neutrophil infiltration may be suitable to attenuate the side effect of IRI in the kidney.

## Oxidative stress and lipid peroxidation

During IRI, the damaged tissue produce excessive amount of ROS cause oxidative stress which changes mitochondrial oxidative phosphorylation, ATP depletion, increase intracellular calcium and activation of membrane phospholipids proteases (41-43). The blood flow during reperfusion phase of IRI can produce oxygen free radicals which leads to lipid peroxidation as main pathway of free radical tissue injuries (44). Formation of free radicals develops renal tissue injury via peroxidation of membrane lipids and oxidative damage of proteins and DNA contribute to apoptosis and cell death (45). Also the down regulation of the antioxidant enzyme system such as catalase, superoxide dismutase, and glutathione peroxidase could be responsible for the pathophysiology of ischemia-reperfusion injury (46). Therefore inhibiting this pathway or prevention of free radical production is the strategy to protect the tissue during IRI.

Studies have shown beneficial effects of free radical scavengers and antioxidants on IRI (47-49). Supplementations with antioxidants agents have protective effects in IRI induced oxidative stress (50-52). Oxygen free radical-mediated renal damage during the reperfusion period following ischemia was prevented by free radical scavengers and antioxidants activity of melatonin (53,54). In addition, inhibition of sympathetic nerve and decrease of catecholamine release (55) may be other mechanisms that melatonin protects renal against IRI. Ulinastatin a potent protease inhibitor with antioxidant activity attenuate renal injury after ischemia by inhibiting apoptosis and neutrophils infiltration (56). Propofol with antioxidant activity reduce IRI (57-59) through reduced lipid peroxidation, cytokines production, increased superoxide dismutase levels and up-regulation of bone morphogenetic protein-2 (BMP2) family that play important roles in diverse cell types (60-62). BMP2 down-regulation in IRI may contribute to an imbalance between cell proliferation and apoptosis thereby causing renal injury (63).

Recent attentions to herbal products encourage scientists to investigate natural agents on IRI. Most of these products exert renoprotective effects on IRI by the radical scavenging and antioxidant activities such as picroliv, an antioxidant extract of *Picrorhiza kurroa* (49), naringin, a bioflavonoid (64) and aqueous garlic extract (65). As conclusion, it is obvious that antioxidants agents could easily act against oxidative stress to protect the tissue against ischemia during IRI phase. However special attention is needed to use the dose of antioxidants, because some antioxidants showed toxic effects in particular dose (66,67).

## Mitochondrial dysfunction

During ischemia, mitochondrial oxidative phosphorylation is suppressed by lack of oxygen. This phenomena impaired ATP synthesis and diminished activity of cellular energy-dependent processes which could contribute to cell death. Mitochondria are the major source of intracellular ROS, and are also the primary target for ROS. ATP depletion stops pumping calcium out of the cell by Na/Ca^2+^antiporter channel therefore calcium accumulate in the cell and sodium accumulated within the cell cannot be removed by Na/K/ATPase (68). In addition intracellular calcium overload occurs from calcium redistribution of endoplasmic reticulum stores (69). Increased cytosolic calcium can activate calcium-dependent phospholipase A2, endonuclease and proteases within the cell that begin cell apoptosis (69,70). In the postischemic cell, mitochondria will be exposed to large amounts of Ca^2+^ and oxygen free radicals. These factors likely contribute to the progressive functional deterioration of mitochondria during the reperfusion phase (71). Therefore the ideal drug therapy needs to be targeted to mitochondria. A number of approaches have been used for targeted delivery of therapeutic agents to mitochondria and should demonstrate very powerful antioxidative properties inside the mitochondria under conditions of oxidative stress such as IRI (72-74). Gentamicin an antibacterial drug induce defect on mitochondrial oxidative phosphorylation and ATP/ADP ratio in reperfusion and therefore it causes renal damage in reperfusion phase more than ischemic phase (75-77).

## Nitrite and nitric oxide

Nitric oxide (NO) the endothelial cell product plays an important role in blood circulation. The half-life of NO in circulation is very short which limited its direct measurement, therefore its metabolites; nitrite and nitrate usually are measured. NO in low concentrations considered as renoprotective against renal ischemia due to its vasodilatory, antioxidant and anti-inflammatory properties, as well as its beneficial effects on cell signaling and inhibition of nuclear proteins (78-80).

Renal IRI activate nitric oxide synthase (NOS) and increase the expression of NOS proteins (81). There are three different isoforms of NOS; endothelial NOS (eNOS) and neuronal NOS (nNOS) produce NO in short bursts in low concentrations for physiological purposes and inducible form of NOS (iNOS) which produces NO in high concentrations. It has been suggested that NO produced by iNOS is a toxic agent whereas eNOS is seen as a protective enzyme (82-84). NO produced in the renal proximal tubules in response to ischemic injury is mediated by iNOS (85).

Studies have suggested that increased NO via iNOS activity during renal ischemia is deleterious to the kidney and inhibition of iNOS before IRI has a dramatic functional protection of kidneys against ischemic renal injury (86). Treatment with sildenafil citrate and tadalafil (phosphodiesterase type 5 inhibitors) decreased lipid peroxidation and myeloperoxidase (indicator of polymorphonuclear infiltration) in renal tissues via inhibition of iNOS expression (87). In addition, ischemia itself can provide endothelial dysfunction, and disturb formation of NO endothelial form of eNOS (88).

The anion nitrite is an end product of NO metabolism (89). In hypoxia and ischemia conditions nitrite convert to NO by NOS and xanthine oxidase enzymes (90). Thus nitrite stored during normoxia and convert to NO in hypoxia conditions can be considered a NO buffer. Several recent reports have demonstrated NO and nitrite-mediated cytoprotection in IRI models (91-94). In addition of NO-dependent cytoprotection, nitrite may act via independent pathway (94,95).

## Renin-angiotensin system

Renin-angiotensin system (RAS) activation and angiotensin II (AgII) level elevation are the important risk factors in IRI (96,97). AgII make renal injury through constrict of renal vessels, enhance vascular sensitivity to sympathetic nerve stimulation (98), cause oxidative stress (99,100) and apoptosis induction (101).

RAS modulate inflammation in renal tissue with two opposite arms effects: angiotensin-converting enzyme (ACE)/AgII/AT1 receptor and angiotensin-converting enzyme 2 (ACE2)/(Ag-(1-7)/Mas receptor (deleterious and protective effect respectively) (102,103). Renal ischemia appears to change the balance of RAS axis (104). Administration of the Mas receptor agonist, AVE0991, attenuated renal tissue damage and infiltration of leukocytes in the kidney IRI (105). ACE2 is a modulator of AgII levels and it convert AgII to Ag-(1-7) in renal tissue and antagonize many deleterious effects of AgII (106,107). New therapeutic strategy is led to activation of ACE2/Ag-(1-7)/Mas axis in renal IRI. Studies have demonstrated that angiotensin converting enzyme inhibitors (ACEIs) and angiotensin receptor blockers have protective effects on IRI in the kidney (108,109). Aliskiren (rennin inhibitor) can directly decrease rennin plasma activity and AgII level (110), has a protective effect on renal IRI through inhibition of RAS, oxidative stress and enhance the anti-apoptosis activity (111).

## Complement system

Many studies have shown the activation of complement system in various IR organs (112-116). Complement system activation releases a number of biologically active products (C4a, C3a, and C5a, C5b-9 and the anaphylatoxins) with proinflammatory and upregulation of adhesion molecules activity (117,118). C5a and C5b-9 have been shown to stimulate endothelial cell expression of selectins and intercellular adhesion molecule-1 (119,120). The recognition of the involvement of complement has led to novel strategies to modulate IRI. Specific C5a receptor antagonist have shown protective effects against renal IRI (121). Also an interfering RNA (siRNA) that target complement 3 (C3) and caspase 3 genes reduced renal IRI (122). Zhou et al. in mice which deficient in C3, C4, C5, and C6 showed that C5b-9 mediated tubular cell damage was etiologic in IRI (123).

## Ischemic preconditioning

Ischemic preconditioning (IP) is a tolerance or adaptability of organ or tissue after primary exposure to a brief ischemia stimulus (124). The kidney has the ability to be preconditioned by a non-lethal period of ischemia, which makes it tolerate to subsequent ischemia-induced injury (125). In studies, renal IP was reduced cell lysis, apoptosis and lipid peroxidation with improvement of renal function in ischemic kidney (126). Reduction of adhesion molecules and inflammatory responses may be the mechanism of IP preventing effects (127,128). But In other studies, ischemic preconditioning appears to be mediated via pre-ischemic activation of adenosine receptors, specifically A_1_ adenosine receptors (129-131).

## Conclusion

Renal injury associated with ischemia/reperfusion results from a dynamic process involving inflammation and some mediators in a complex interaction. Formation of oxidative stress and lipid peroxidation seems to be major factors which promotes the inflammation process during IRI. A better understanding of the pathophysiology and therapeutic approaches underlying the functional defects found in ischemic acute renal failure will also require that we bear in mind the complexity of illness.

## Authors’ contribution

MM conducted literature review and wrote the article. MN planned and conducted literature review, and finalized it. All authors read and signed the manuscript.

## Conflicts of interest

The authors declared no competing interests.

## Funding/Support

None.
